# Role of Liquid-Based Cytology in the Endoscopic Diagnosis of Pancreatic Ductal Adenocarcinoma

**DOI:** 10.3390/jcm13206148

**Published:** 2024-10-15

**Authors:** Koh Kitagawa, Akira Mitoro, Hisae Suzuki, Fumimasa Tomooka, Shohei Asada, Jun-Ichi Hanatani, Yuki Motokawa, Tomihiro Iwata, Yui Osaki, Maiko Takeda, Hitoshi Yoshiji

**Affiliations:** 1 Department of Gastroenterology, Nara Medical University, Nara 634-8522, Japan; 2 Division of Endoscopy, Nara Medical University, Nara 634-8522, Japan; 3 Department of Diagnostic Pathology, Nara Medical University, Nara 634-8522, Japan

**Keywords:** cytology, endoscopic ultrasound-guided fine needle aspiration, endoscopic ultrasound-guided fine needle biopsy, endoscopic ultrasound-guided tissue acquisition, endoscopic retrograde cholangiopancreatography, histology, liquid-based cytology, pancreatic ductal adenocarcinoma

## Abstract

Recently, endoscopic ultrasound-guided tissue acquisition (EUS-TA) has been widely used to diagnose pancreatic ductal adenocarcinoma (PDAC). The histological examination of core tissues acquired using novel biopsy needles is the primary diagnostic approach for patients with PDAC. However, in patients with early-stage PDAC, such as Stages 0 and I, EUS-TA can be challenging, and its diagnostic accuracy may be limited. This presents a clinical dilemma: The earlier that clinicians attempt to accurately diagnose PDAC, the more difficult it becomes to do so using EUS-TA. Liquid-based cytology (LBC) is a technique for preparing pathological specimens from liquefied cytology specimens by placing the collected material in a special fixative preservative fluid. LBC offers advantages, such as specimen optimization with reduced blood interference, a high cell-collection rate, and the simplicity of the procedure in the endoscopy room. The use of LBC may improve diagnostic accuracy, particularly for early-stage PDAC. Therefore, we emphasize that cytology remains a valuable tool for the endoscopic diagnosis of PDAC. In this review, we discuss the role of LBC in the endoscopic diagnosis of PDAC.

## 1. Introduction

Endoscopic ultrasound-guided tissue acquisition (EUS-TA) is a widely used method for the pathological diagnosis of pancreatic ductal adenocarcinoma (PDAC) and enlarged lymph nodes because of its high diagnostic accuracy and low adverse event rate. Recent advances in puncture needle technology have enabled the retrieval of large core tissue samples, making histologic examination the mainstay tool for diagnosing PDAC [1]. Recent studies have also explored the use of comprehensive genomic profiling on samples obtained by EUS-TA [2,3]. However, a prospective study by Hisada et al. [3] reported a relatively high adverse event rate of 9% when EUS-TA was used for comprehensive genomic profiling with a 19-gauge fine needle biopsy (FNB) needle. Additionally, diagnosing small lesions such as Stage 0/I PDAC using EUS-TA can be challenging [4,5,6,7], leading to reliance on cytology in some cases. Furthermore, the usefulness of cytology via conventional endoscopic retrograde cholangiopancreatography (ERCP) for early-stage PDAC diagnosis has recently been reported [6,8,9,10]. Therefore, cytology continues to play a significant role in the endoscopic diagnosis of PDAC [11].

Conventional cytologic diagnosis using EUS-TA, in which the specimen is directly smeared onto a glass slide, has been reported to have a sensitivity ranging from 64% to 96% for the diagnosis of malignant pancreatic mass lesions [12,13,14,15,16]. The conventional smear method offers the advantage of rapid on-site evaluation (ROSE), where specimens are fixed and stained in situ, allowing immediate microscopic evaluation to assess adequacy and provide a rapid diagnosis. ROSE improves the rate of positive diagnoses and minimizes the number of needle passes required [14]. However, the availability of facilities capable of performing ROSE is limited due to the requirement for on-site specimen processing, specialized equipment, and trained personnel as well as the time needed for each puncture.

Alternatively, liquid-based cytology (LBC) is a method for preparing pathological specimens by placing liquefied cytology specimens in special preservative fluids. The usefulness of this method in cervical cancer screening is well documented and is gaining popularity [17]. Recent studies have explored the usefulness of LBC for analyzing various nongynecological masses, including head and neck, thyroid, breast, lymph node, and pancreatic masses [17,18,19,20,21]. Although LBC may be difficult to apply with ROSE, it offers easy specimen processing in the endoscopy room and a high cell collection rate. In addition, the efficacy of LBC for pancreatic juice cytology (PJC) via conventional ERCP has been demonstrated [10,22], showing potential to overcome the limitations of cytology via ERCP, such as low diagnostic sensitivity due to inadequate specimen processing.

Evaluating tissue structure is challenging in cytological diagnosis. However, even with limited tumor cells collected endoscopically, PDAC can be diagnosed successfully through cellular analysis. This review highlights the role of LBC in the current era, where histology via EUS-TA has emerged as the primary diagnostic tool for PDAC.

## 2. Overview and Characteristics of LBC

LBC involves suspending collected specimens in a preservative fluid with hemolytic and protein-solubilizing properties, homogenizing them, and then smearing them in a thin layer on a glass slide. In the 2000s, LBC was introduced for cervical cancer screening because of its compatibility with automated screening machines and its advantages, such as fewer inadequate specimens by eliminating blood, mucus, and inflammatory background, as well as achieving a high cell-collection rate [23]. More recently, LBCs have been used for cytological diagnosis in various areas, including the uterine body, sputum, urine, body fluids, and puncture aspiration specimens from organs such as the mammary gland, thyroid, lymph nodes, and pancreas, in addition to the cervix.

## 3. Types of LBC

There are several methods of LBC, which depend on the composition of the preservative fluid and the method of sample preparation. The most commonly used LBC methods are ThinPrep (Hologic Inc., Marlborough, MA, USA) and SurePath (BD Diagnostics, Burlington, NC, USA). Both preservative fluids are alcohol buffered, but each has various innovations to preserve cell morphology and clean background via hemolytic action.

ThinPrep uses a filter transfer method in which negative pressure is applied to the filter to remove contaminants from the liquefied sample via aspiration. Positive pressure was then applied to smear the target cells onto a glass slide. This method requires automated filter-based smear processing and does not allow manual sample preparation, which necessitates equipment investment. SurePath is a centrifugal sedimentation method. After centrifugation to remove background material such as mucus from the liquefied sample and collect primarily target epithelial cells, the sample is resuspended. Gravity causes cells to spontaneously settle on a glass slide. A dedicated glass slide with a center charged to “+” is used to adsorb and smear cancer cells with a surface charged to “−” [24]. This potential difference facilitates efficient tumor cells adsorption on glass slides.

## 4. The Specimen-Handling Flow in the EUS-TA/ERCP

**Figure 1:**
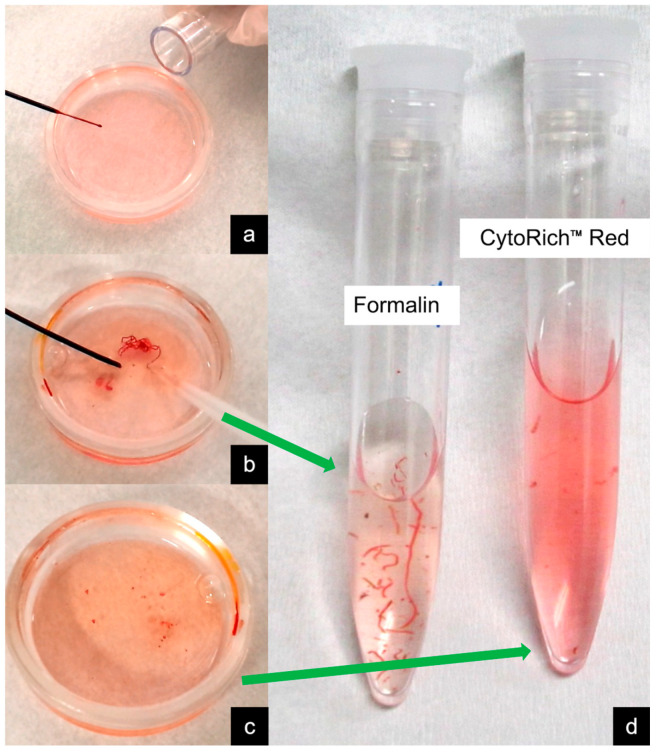
The specimen-handling flow in endoscopic ultrasound-guided tissue acquisition (EUS-TA) at our institution. (**a**) A small amount of dedicated preservative fluid (BD CytoRich Red Preservative; Becton Dickinson Japan, Tokyo, Japan) is placed in a Petri dish. (**b**) The specimen obtained by EUS-TA is pushed out of the puncture needle and promptly mixed directly with the preservative fluid. (**c**,**d**) Pieces of tissue with an apparent bulk are aspirated using a syringe, fixed in formalin, and prepared for histology. The residual paste or liquid specimens with preservative fluid are adapted to liquid-based cytology.

**Figure 2:**
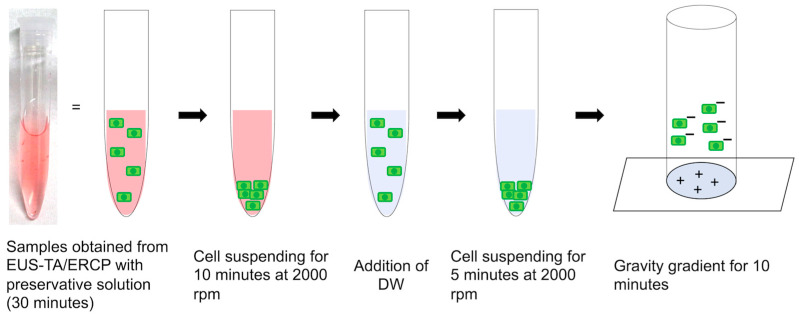
Laboratory protocol for processing samples from endoscopic ultrasound-guided tissue acquisition (EUS-TA) and endoscopic retrograde cholangiopancreatography (ERCP) at our institution.

**Figure 3:**
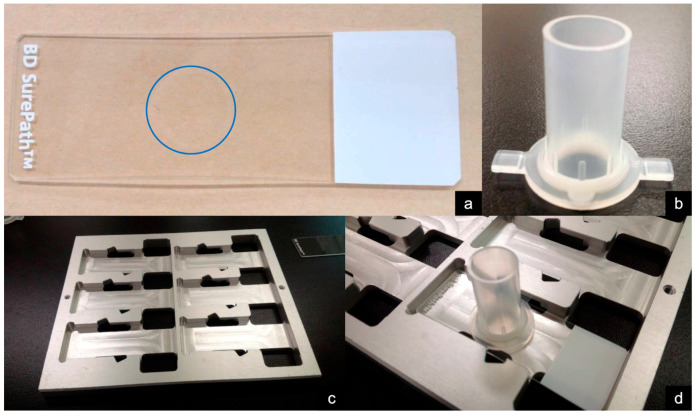
Dedicated instruments for liquid-based cytology (SurePath method). (**a**) Dedicated glass slide (BD SurePath PreCoat slides; Becton Dickinson, Japan), with a charged center marked with a “+” (blue circle). (**b**) Settling chamber. (**c**) Tray for glass slides. (**d**) Dedicated glass slide and chamber mounted on trays.

**Figure 4:**
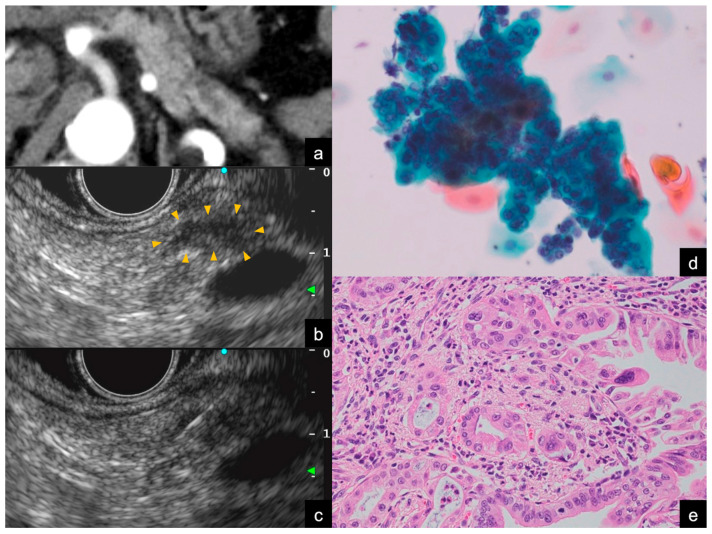
Findings of Stage I (T1) pancreatic ductal adenocarcinoma. (**a**) Contrast-enhanced computed tomography (CE-CT) image. A pancreatic duct stenosis was observed in the tail of the pancreas, with dilation of the caudal pancreatic duct. Focal atrophy of the pancreatic parenchyma was noted at the site of the pancreatic duct stenosis, but no mass was visible on this CT. (**b**,**c**) Endoscopic ultrasound (EUS) images. A small hypoechoic mass less than 10 mm in diameter was identified at the site of the pancreatic duct stenosis (orange arrowheads). EUS-guided tissue acquisition was performed with a conventional 25-Gauge needle for fine needle aspiration. (**d**) Images of liquid-based cytology of specimens obtained from EUS-TA (Papanicolaou staining). The background of inflammatory cells and artifacts has been removed, revealing solitary, scattered tumor cells that can be evaluated. The cytological diagnosis was positive for adenocarcinoma. (**e**) Distal pancreatectomy was performed. Histopathologic findings revealed a 10 mm invasive carcinoma localized within the pancreas (hematoxylin–eosin staining).

Figure 1 illustrates the specimen-handling flow in EUS-TA at our institution. The specimens collected by EUS-TA were promptly mixed with a preservative fluid (BD CytoRich Red Preservative; Becton Dickinson Japan, Tokyo, Japan). Prompt mixing minimizes sample denaturation and enhances cell-collection efficiency, although it precludes ROSE. The preservative fluid contains ammonium chloride, ammonium oxalate, ethanol, methanol, isopropanol, formalin, and ethylene glycol. This fluid lyses red blood cells, solubilizes proteins, and preserves diagnostically relevant materials. Figure 2 and Figure 3 depict the specimen-processing protocol at our hospital. For PJC via ERCP, the pancreatic juice was initially centrifuged, and the supernatant was discarded. The sediments were then suspended in preservative fluid and processed similarly to EUS-TA specimens. Finally, negatively charged cancer cells adhere to the positively charged surface of glass slides (BD SurePath PreCoat slides; Becton Dickinson, Japan). After washing with distilled water, a homogeneous thin cell layer with a clear background was obtained over a limited area of the slide (Figure 4).

Samples are immersed in the preservative fluid for at least 30 min. The supernatant is then discarded, and distilled water (DW) is added. The solution is then centrifuged at 2000 rpm for 5 min. The supernatant is discarded, and DW is added once more. The solution is resuspended and placed in a chamber affixed to a glass slide with a positive charge at the center.

## 5. Advantages and Disadvantages of LBC

The advantages of the LBC method over conventional smear methods include the following:

1. Reduction in inadequate specimens by removing blood and mucus.
2. High cell-collection rate.
3. Uniform specimens with less cell overlap.
4. Reduced viewing time under a microscope as target cells are localized on the slide.
5. Reduction in labor and examination time in the endoscopy room.
6. Standardization of specimen processing for cell collection and specimen preparation.
7. Cells in the preservative fluid remain stable for several weeks and can undergo immunostaining and molecular biological analysis if necessary.
8. Liquefied samples can be processed by outsourcing to laboratories.
9. Automated screening machines can be used.

In cytology via EUS-TA/ERCP for pancreatic masses, the advantages of points (1) and (2) are particularly significant. In our experience, since the introduction of EUS-TA, no inadequate specimens were found after an average of 2.1 punctures, mainly using 25-gauge FNA needles, in 222 lesions [25].

Alternatively, the disadvantages of the LBC method compared with the conventional smear methods include the following:

1. Changes in cell morphology due to different fixation and smear methods.
2. Loss of background information necessary for diagnosis.
3. Complicated procedures in the pathology department.
4. Increased cost.

Although points (1) and (2) can be challenging when diagnosing with LBC, they do not significantly impact diagnostic performance once pathologists and cytotechnologists are familiar with the cytologic characteristics of the LBC specimen [26]. However, the diagnostic performance of LBC for pancreatic tumors other than PDAC, such as neuroendocrine and metastatic pancreatic tumors, may be inferior [27]. In addition, LBC requires a high initial investment and consumable costs. In particular, ThinPrep is mainly suitable for large facilities because of its high initial investment and maintenance costs, which makes it less accessible for smaller facilities. In contrast, SurePath requires only a centrifuge and inexpensive consumables, making it more feasible for use in smaller facilities.

## 6. Diagnostic Yields of LBCs in EUS-TA of Pancreatic Tumors

Reports on the diagnostic performance of LBC by EUS-TA for pancreatic tumors are summarized in Table 1. The earliest study was a retrospective analysis conducted in 2004 by de Luna et al. [28], in which 67 pancreatic tumors were evaluated using the ThinPrep method. The sensitivity for the diagnosis of PDAC was 58%, lower than that of the conventional smear method. In addition, a marked difference in cytologic characteristics was observed between specimens processed using ThinPrep and the conventional smear method, suggesting the need for modified diagnostic criteria when using the ThinPrep method. They also attributed the low diagnostic sensitivity to the use of split specimens in ThinPrep slide preparation, with residual samples from multiple direct smears used. Subsequent prospective studies using the ThinPrep method also reported low LBC sensitivity [29,30,31], consistent with the findings of de Luna et al. Lee et al. highlighted LBC’s ability to accurately diagnose two out of three false-negative cases of malignancy that were challenging to diagnose using the conventional smear method due to heavy blood contamination [30].

In 2017, Hashimoto et al. reported the diagnostic yield of the SurePath method for pancreatic specimens obtained by EUS-TA [27]. They conducted a retrospective analysis to compare the diagnostic value of LBC with that of conventional smear methods using propensity score matching. The findings revealed that LBC using the SurePath method was more sensitive compared to the conventional smear method for diagnosing PDAC. Subsequent studies with larger sample sizes [25,32,34,35,37] have confirmed the superiority or noninferiority of LBC using the SurePath method [27,34,39]. Since the first report on LBC in 2004, Chun et al. conducted a randomized controlled trial comparing LBC using the SurePath method with the smear method [35] and found no difference in diagnostic sensitivity for PDAC between the two groups. However, LBC had lower specimen inadequacy rates, less blood contamination, and preserved tumor cytomorphologic features on slides. These findings indicate that LBC is more justified compared to the conventional smear method for EUS-TA. It is noteworthy that recent studies have demonstrated a good diagnostic performance of LBC without ROSE. Our previous study showed the successful diagnosis of PDAC using LBC with SurePath and without ROSE [25]. In facilities where ROSE is not available, LBC may be a viable option to improve the diagnostic accuracy of EUS-TA [11].

However, most previous reports on EUS-TA using the LBC method employed conventional FNA needles and the traditional dry-suction method for tissue sampling. Advanced FNB needles with superior puncture ability are now available, and investigating the diagnostic capability of LBC in EUS-TA using these needles remains necessary. When employing the conventional dry-suction method, the stylet is removed and an air-filled, prevacuum 10- or 20-mL syringe is attached to the proximal end of the needle and opened once inside the lesion to apply negative pressure. When using the wet-suction technique, the needle is flushed with saline to replace the air column and a prevacuum syringe is used for suction. For the slow-pull technique, tissue sampling is performed by applying minimal negative pressure as the stylet is slowly and continuously retracted. These suction techniques aim to reduce blood contamination during EUS-TA with FNB needles and to obtain higher-quality specimens [40,41]. These differences in suction techniques may also impact the diagnostic performance of EUS-TA with LBC.

Furthermore, cost-effectiveness is a crucial factor in modern healthcare. Therefore, further studies on the cost-effectiveness of LBC are required. However, in the SurePath method, the specialized instruments shown in Figure 3 are inexpensive. Although disposable, the dedicated glass slides (Figure 3a) are not costly. Additionally, the dedicated settling chamber (Figure 3b) and tray (Figure 3c) are reusable. Consequently, the cost of LBC using the SurePath method does not increase substantially compared with the conventional smear method.

## 7. Diagnostic Yield of LBC in ERCP for Pancreatic Tumors

**Figure 5:**
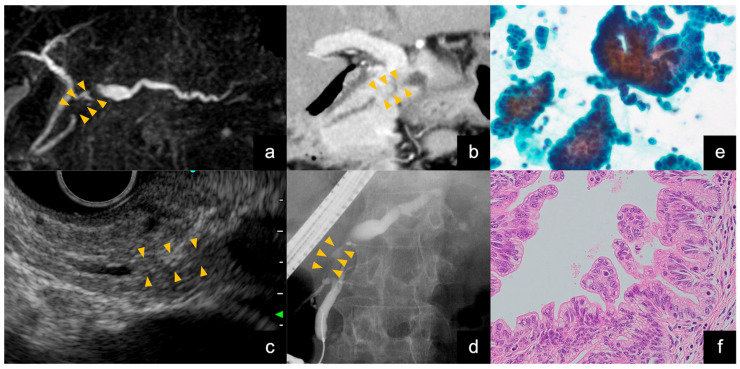
Findings of Stage 0 pancreatic ductal adenocarcinoma. (**a**) Image of magnetic resonance cholangiopancreatography. Pancreatic duct stenosis was found in the head of the pancreas (orange arrowheads), and the caudal pancreatic duct was dilated. (**b**) Contrast-enhanced computed tomography (CE-CT) findings. There was pancreatic duct stenosis in the head of the pancreas (orange arrowheads), and the caudal pancreatic duct was dilated. However, no mass was visible on this CT scan. (**c**) Endoscopic ultrasound (EUS) image. No mass was visible at the site of pancreatic duct stenosis, even on EUS (orange arrowheads). (**d**) Endoscopic retrograde cholangiopancreatography. Pancreatography revealed stenosis of the main pancreatic duct in the head of the pancreas (orange arrowheads). An endoscopic nasopancreatic drainage tube was placed, and serial pancreatic juice aspiration cytology was performed. (**e**) Images of liquid-based cytology obtained via pancreatic juice cytology (Papanicolaou staining). The background of inflammatory cells and artifacts is removed, and solitary, scattered tumor cells can be evaluated. The cytological diagnosis was positive for adenocarcinoma. (**f**) A pancreatoduodenectomy was performed. Histopathologic findings on the resected specimen showed atypical columnar epithelial cells with chromatin-enriched enlarged nuclei proliferating in a hypopapillary fashion into the lumen of the pancreatic duct (hematoxylin–eosin staining). There was no obvious stromal invasion, and the diagnosis of carcinoma in situ was made.

The diagnostic value of EUS-TA for solid pancreatic tumors is well established. However, performing punctures on small lesions can be technically challenging. Recent advancements in diagnostic imaging have increased the number of carcinoma in situ (CIS) cases detected in the presence of minor pancreatic duct strictures [8]. Notably, CIS cannot be diagnosed using EUS-TA because it does not form a mass. For Stage 0/I PDAC [42], diagnosis via EUS-TA is challenging and conventional pathological diagnostic approaches such as ERCP are still relied upon (Figure 5).

Cytologic diagnosis of PDAC via ERCP includes pancreatic duct brush cytology and PJC. In recent years, the diagnostic utility of serial pancreatic juice aspiration cytology, in which multiple PJCs are performed sequentially with endoscopic nasopancreatic drainage tube placement, has been reported, particularly in Japan [8,9,10]. This method allows for the diagnosis of early-stage PDAC, including CIS without mass formation, which is difficult to diagnose with EUS-TA. However, the diagnostic yield of PJC may be inferior to that of EUS-TA because of the lower number of cells collected.

Table 2 presents the diagnostic utility of LBC via ERCP for pancreatic tumors. Miyamoto et al. reported 90 cases of suspected malignant intraductal papillary mucinous neoplasm (IPMN) that were difficult to diagnose using EUS-TA. They found that the diagnostic accuracy of LBC in PJC obtained via ERCP was higher than that of the conventional smear method [22]. The sensitivity and accuracy were 40% and 76%, respectively, with LBC identified as a significant factor contributing to improved diagnostic accuracy in multivariate analysis. Particularly, the accuracy of LBC was significantly higher than that of the smear method in IPMN patients with “worrisome features” (PJC accuracy: smear 66% vs. LBC 93%). Miyamoto et al. attributed the high diagnostic performance of the SurePath method to its ability to efficiently remove mucus, blood, and inflammatory cells from PJC specimens of patients with IPMN. Alternatively, Kenyon et al. reported that BD SurePath did not reduce cellularity after the addition of mucus, whereas ThinPrep significantly decreased cellularity [43] due to direct occlusion of the filtration membrane by excess mucus. Miyamoto et al. concluded that the SurePath method is better suited for the diagnosis of IPMN.

Furthermore, we have reported the excellent diagnostic yield of LBC in PJC of PDAC [10]. In this study, serial pancreatic juice aspiration cytology via endoscopic nasopancreatic drainage was performed in 24 patients with suspected PDAC that were difficult to diagnose using EUS-TA. The diagnostic sensitivity and accuracy of PDAC were 81.8% and 91.7%, respectively. Notably, three out of the 24 patients in this study were found to have Stage 0 PDAC, which is equivalent to CIS.

Kanno et al. reported the details of 200 cases of Stage 0–1 PDAC in Japanese patients [44]. In their study, only 125 cases (62.5%) were confirmed to have malignant tumors through cytological diagnosis prior to resection. In the remaining 75 cases (37.5%), pancreatic ductal adenocarcinoma was strongly suspected based on abnormal findings, such as MPD dilatation detected via various imaging methods, and the decision to resect was made after obtaining informed consent. Furthermore, most Stage 0 cases were diagnosed using SPACE, with only one case diagnosed using EUS-TA. Compared to this prior report, our LBC results for diagnosing early-stage PDAC are superior. However, because our case numbers are low, further multicenter studies are needed.

## 8. Genetic Analysis of Residual LBC Samples

Genetic analysis of residual LBC specimens obtained via EUS-TA of pancreatic masses has been conducted in a limited number of studies in the literature. The *K-ras* mutation is the most common genetic mutation in patients with PDAC [45] and is considered the most “RAS-addicted” cancer.

Cytotechnologists and pathologists at our institution have successfully performed *K-ras* mutation analysis using residual LBC specimens [46]. In this study, *K-ras* mutation analysis using residual LBC samples yielded positive results in all cases. The combined analysis of cell block (CB) and *K-ras* mutation improved the sensitivity and diagnostic accuracy of PDAC. The sensitivity, specificity, and accuracy of CB examination alone were 77.4%, 100%, and 81.3%, respectively. When CB examination was combined with *K-ras* mutation analysis, the values improved to 90.3%, 92.3%, and 90.7%, respectively. Furthermore, *K-ras* mutations were detected in 8 (57.1%) of 14 PDAC samples, indicating inconclusive CB results. Four years later, the authors also reported that next-generation sequencing targeting six genes was successful in 84.6% of patients (44/52) [47]. Among 33 cases of PDAC, *K-ras*, *TP53*, *CDKN2A*, and *SMAD4* mutations were identified in 31 (94%), 16 (48%), three (9%), and two (6%) cases, respectively. Of the 11 benign cases, only one *K-ras* mutation was identified. Based on next-generation sequencing results, 18 out of 33 PDAC cases (55%) were classified as highly dysplastic or more, whereas 10 out of 11 benign lesions were classified as non-malignant, consistent with the final diagnosis.

Furthermore, Itonaga et al. reported a successful *K-ras* mutation analysis rate of 98.6% (274/278) in residual LBC specimens. *K-ras* status was found to be correlated with response to chemotherapy and predicted prognosis in patients with unresectable PDAC [48]. Knowledge of the *K-ras* status at the initiation of therapy could help identify mutations that require targeted therapy or inclusion in clinical trials, particularly in cases of wild-type *K-ras* PDACs.

These findings support the validity of genetic analysis of residual LBC specimens obtained via EUS-TA for pancreatic masses. However, the current analysis focused on a limited number of genes, which may pose challenges for comprehensive genetic analysis. The further development of panel tests targeting key gene mutations could facilitate widespread genetic analysis using residual LBC specimens. Further studies on a larger number of genes are required to advance this approach.

## 9. Conclusions

A clinical dilemma arises when clinicians attempt to accurately diagnose PDAC at an early stage, as it becomes more difficult to diagnose the disease using EUS-TA. LBC enables the capture of representative cells from the entire specimen on a cytology slide, making the diagnosis of PDAC possible even in cases with few cancer cells. Advances in imaging studies have improved the detection rate of early-stage PDAC. Therefore, we emphasize that cytology remains a valuable tool for the endoscopic diagnosis of PDAC.

## Figures and Tables

**Table 1:** Diagnostic yields of liquid-based cytology (LBC) in ultrasound-guided tissue acquisition for pancreatic tumors.

Authors	Year	Study Design	Sample Size	LBC Technique	Rapid On-Site Evaluation Available	Puncture Needle	Suction Technique	Sensitivity (%)	Specificity (%)	Comparison with Smear Cytology (SC)
de Luna et al. [28]	2004	Retrospective	67	ThinPrep	Yes	FNA	ND	58	100	LBC < SC
LeBlanc et al. [29]	2010	Prospective	50	ThinPrep	Yes	FNA	ND	62	100	LBC < SC
Lee et al. [30]	2011	Prospective	58	ThinPrep	No	FNA	Dry suction with 10-mL syringe	75	100	LBC < SC
Qin et al. [31]	2014	Prospective	72	ThinPrep	No	FNA	ND	73	100	LBC = SC
Hashimoto et al. [27]	2017	Retrospective	126	SurePath	No	FNA	Dry suction with 10–20-mL syringe	96.6	100	LBC > SC
Yeon et al. [32]	2018	Prospective	75	SurePath	No	FNA	Dry suction with 10-mL syringe	61	100	LBC < SC
Itonaga et al. [33]	2019	Retrospective	204	ThinPrep	Yes	FNA	Dry suction with 20-mL syringe	93.2	100	LBC + SC > SC
Mitoro et al. [25]	2019	Retrospective	222	SurePath	No	FNA	Dry suction with 10-mL syringe	93.9	95.1	No
Zhou et al. [34]	2020	Retrospective	514	SurePath	No	FNA	Dry suction with 10-mL syringe and slow-pull	71.4	100	LBC > SC
Chun et al. [35]	2020	Randomized controlled trial	170	SurePath	No	FNA	Dry suction with 10-mL syringe	88	100	LBC = SC
Choi et al. [36]	2021	Prospective	63	ND	No	FNA	Dry suction with 10-mL syringe and slow-pull	73.8	100	No
Tomita et al. [37]	2021	Retrospective	46	SurePath	No	FNA	Slow-pull	85.4	100	No
Huang et al. [38]	2021	Retrospective	52	ThinPrep	Yes	FNA	ND	86.8	100	LBC = SC
Bürger et al. [39]	2022	Retrospective	172	ND	No	FNA	Dry suction with 10-mL syringe	87.8	100	LBC > SC

LBC, liquid-based cytology; SC, smear cytology, FNA, fine needle aspiration; ND, not described.

**Table 2:** Diagnostic yield of LBC in ERCP for pancreatic tumors.

Authors	Year	Study Design	Sample Size	Disease	LBC Technique	Cytology Samples	Sensitivity (%)	Specificity (%)	Accuracy (%)	Comparison with SC
Miyamoto et al. [22]	2020	Retrospective	90	Malignant IPMN	SurePath	PJC	40	100	76	LBC > SC
Kitagawa et al. [10]	2022	Retrospective	24	PDAC	SurePath	PJC	81.8	100	91.7	No

LBC, liquid-based cytology; SC, smear cytology; ERCP, endoscopic retrograde cholangiopancreatography; IPMN, intraductal papillary mucinous neoplasm; PDAC, pancreatic ductal adenocarcinoma.

## Data Availability

Not applicable.
